# Supplementary material for: Rapid turnover of life-cycle-related genes in the brown algae
Source: Genome Biol. 2019 Feb 14;20:35. doi: 10.1186/s13059-019-1630-6 (PMC6374913; doi:10.1186/s13059-019-1630-6)
Supplement: Supplementary file 3 — Protein alignments, corresponding Gblocks html file and CDS sequences for the lineage conserved gametophyte-biased genes. (ZIP 2109 kb) [file 13059_2019_1630_MOESM3_ESM.zip › Con_GA_alignments/Ec-07_003910.aa_ali.fasta-gb.htm]

Ec-07\_003910.aa.fasta.trans.aa\_ali.fasta


## Gblocks 0.91b Results

Processed file: **Ec-07\_003910.aa.fasta.trans.aa\_ali.fasta**  
Number of sequences: **2**  
Alignment assumed to be: **Protein**  
New number of positions: **436** (selected positions are underlined in blue)

```
                         10        20        30        40        50        60
                 =========+=========+=========+=========+=========+=========+
Ec-07_003910     -----------------------MVAADMGHLKDTVEKLDHIVRETMGQHGKDLAAVLVQ
MSTRG.23000.1    XNSEAAEQRQHHGLSLSNPRAFELVTADMGHLKDTVEKLDLAFRETMGQQGRDLAAVMDQ
                                         ####################################


                         70        80        90       100       110       120
                 =========+=========+=========+=========+=========+=========+
Ec-07_003910     LGLERPSRSNLVAGSESRPMPAEG-------LTNNKARGATPAWPSPLQQAP--QDQLQL
MSTRG.23000.1    LGVEPAGRHELSTGNSPFPKSAHGHGTNGKENINNEREDERPAWSLPPPKATGHNNQKRL
                 ########################         ##################     ####


                        130       140       150       160       170       180
                 =========+=========+=========+=========+=========+=========+
Ec-07_003910     AGGSRQGQGDASVLGAGGGVDHVAVRGTAEDGSLAGGGPTTGKALGRDTQGNGKGAAGGL
MSTRG.23000.1    SKIAPAARGQEKGSNEQKGSKQAPPSGNDKFARVS-ASAVSLPGSGRAIAASGDVGATGG
                 #########                                    ###############


                        190       200       210       220       230       240
                 =========+=========+=========+=========+=========+=========+
Ec-07_003910     TEEGARGAAEGVAVWNAYQRGKWVNGQWVPKVLQDDTSPDLSQLATTGKKQRRVLLLSEK
MSTRG.23000.1    VGEPTKN---AGAVWNAYQRGKWVNGQWIPKVLQDDTSPDLSKLASNNEQQRRVLLLSER
                 ###         ################################################


                        250       260       270       280       290       300
                 =========+=========+=========+=========+=========+=========+
Ec-07_003910     RSGGHAVGGVLNADTRVFYVADPCRMGGGPEALDAGACSLAVTRLLACQPTTNDIRNLFS
MSTRG.23000.1    RSGGHAVGNILDADPRVFYVADPCRMGGGPEALDPGACSVAVSRLLACQPTINDIRNLFS
                 ############################################################


                        310       320       330       340       350       360
                 =========+=========+=========+=========+=========+=========+
Ec-07_003910     FSYIVERSRVLSESEGLRWQAEGTPDENDEETRAAVIRQCRLSSVVVVKETRLAEVAPRL
MSTRG.23000.1    FSYIVERSRVLSQFEDLRWKAGGTPEENDEEVRAAVVQQCRLSSVVVVKETRLAEVGSKI
                 ############################################################


                        370       380       390       400       410       420
                 =========+=========+=========+=========+=========+=========+
Ec-07_003910     STPELDLEFLHIVRDPRAAIHGMAELWEKSDNKMERSMARDCSDVASRLCTSAENKLKFL
MSTRG.23000.1    SAEELGTEFLHVVRDPRAAIHGMSERWQSSGNKLEQALAKDCSDMASRVCSSSEKKLKDL
                 ############################################################


                        430       440       450       460       470       480
                 =========+=========+=========+=========+=========+=========+
Ec-07_003910     TLLGKQRFRVLKHEMVTEAIRSTS-------------------MRSSAENSPGTAVA--G
MSTRG.23000.1    TELGKERFRVLKYEMFVEAPFAMAKSVYGWLKLGDFPQEVVDRIQSIPAMGSTQAAQGPS
                 ###################                          ##########     


                        490       500       510       520       530       540
                 =========+=========+=========+=========+=========+=========+
Ec-07_003910     NGSKSTMGGFDWATRWAVEMPTFRRQQVQLACHPLLVALGYDDPEDPEEQQDAGEDGQEE
MSTRG.23000.1    HGTRAALGRFEWATRWAVEMPAFRRQQIQHACQPLLVALGYDEADSFEDGERDADEQEGQ
                  ###############################################            


                        550       560       570       580       590       600
                 =========+=========+=========+=========+=========+=========+
Ec-07_003910     SQGESSSEELSTAFDGTSSDRGAGGTGFGSSMTDGVNRVGHGGGGAEKLRASSALPSSSL
MSTRG.23000.1    GPASSSPEPDAARFGSVEGAAPSRGQGWRASASASSSLAAP----VKEVKTAAGAGAAAI
                     ##########                                              


                        610       620       630
                 =========+=========+=========+=
Ec-07_003910     SAATAARNPESVAAAADTILPGPDAIARHYG
MSTRG.23000.1    SGANAS----AAVPADRSAVDGGSVSA----
                               #############
```

```
Parameters used
Minimum Number Of Sequences For A Conserved Position: 2
Minimum Number Of Sequences For A Flanking Position: 2
Maximum Number Of Contiguous Nonconserved Positions: 8
Minimum Length Of A Block: 10
Allowed Gap Positions: None
Use Similarity Matrices: Yes
```

```
Flank positions of the 9 selected block(s)
Flanks: [25  84]  [94  111]  [117  129]  [166  183]  [193  439]  [466  475]  [482  528]  [545  554]  [615  627]  

New number of positions in Ec-07_003910.aa.fasta.trans.aa_ali.fasta-gb:  436  (69% of the original 631 positions)
```
